# Can carbon labels encourage green food choices?

**DOI:** 10.3389/fpsyg.2022.902869

**Published:** 2023-01-27

**Authors:** Swen J. Kühne, Ester Reijnen, Lea Laasner Vogt, Melanie Baumgartner

**Affiliations:** Applied Cognitive Sciences, Psychological Institute, School of Applied Psychology, Zurich, Switzerland

**Keywords:** food product choices, carbon label, CO_2_e emissions, sustainability, label design

## Abstract

**Introduction:**

A conventionally grown kiwi from Spain or an organic pineapple from Ghana? Which is the more environmentally friendly option? Given that the production and distribution of food is responsible for about a quarter of our CO_2_e emissions and thus plays a role in climate change the answer to such questions and, accordingly, making the right food product choices is crucial. The problem, however, is that it is difficult for consumers to calculate the CO_2_e value of food as it depends on several specifications such as the type of food, origin, etc. Could carbon labeling of food circumvent this problem and help consumers make more environmentally friendly choices?

**Methods:**

In an online experiment, 402 participants had to choose 20 food products from a fictitious online shop. The participants were randomly assigned to either one of three food labeling conditions (Star Rating, Green Foot, and Traffic Light Label, short TLL) or the control condition.

**Results:**

The labeling conditions resulted in lower overall CO_2_e emissions, the purchase of more green food products and fewer red food products than in the control condition. The TLL outperformed the other two labels and was also the most accepted.

**Discussion:**

The carbon TLL is therefore a promising intervention to help consumers to not only choose more environmentally friendly foods, but also make a significant contribution to the fight against climate change.

## 1. Introduction

Kiwi or pineapple? Because you care about the environment, you want to choose the product that has the smallest *carbon footprint*. However, for its calculation, one needs several specifications for each product, such as how (e.g., organic) and where (e.g., Switzerland) it was produced, how it was transported (e.g., by air to overseas) and how it was preserved (e.g., canned; Poore and Nemecek, 2018; Rogissart et al., 2019). These specifications must then be weighted according to their importance and summed up. The food with the lower value should then be chosen. Given the *complexity* of this calculation, and to reduce the cognitive effort involved, consumers instead use simple heuristics (e.g., “whether the product comes from the region or not”) to choose between different food options (see Thøgersen et al., 2012, on organic food choices or Scheibehenne et al., 2007, on food choices in general). Nevertheless, research has shown that consumers’ carbon footprint *estimates* are far from accurate, especially for foods with a high carbon footprint, such as meat or dairy products (see Camilleri et al., 2019, or Shi et al., 2018). This has consequences given that 20–40% of our carbon emissions are food related (see Poore and Nemecek, 2018). Improving consumers’ ability to assess the carbon footprint of food is therefore key to encouraging consumers to buy more environmentally friendly food (Feucht and Zander, 2017).

One way to achieve this is through the introduction of *carbon labels*, especially since this is also widely supported by consumers (Carbon Trust, 2020). Therefore, in the recent years, different types of carbon labels have been developed and launched on the market. For example, the Eco Label index^1^ alone already lists 455 different eco labels, of which about 35 are carbon labels (and there are still some missing from this list). To distinguish between the different types of carbon labels, several classification systems have been proposed (see Schaefer and Blanke, 2014; Thøgersen and Nielsen, 2016; Meyerding et al., 2019; Lemken et al., 2021; Taufique et al., 2022). Taufique et al. (2022), for example, suggested a classification into 4 types of carbon labels: *certificate*, *ordinal rating*, *quantitative* and *ordinal plus quantitative rating* labels.

## 2. Background literature on carbon labels

### 2.1. Types of existing carbon labels

**Figure 1:**
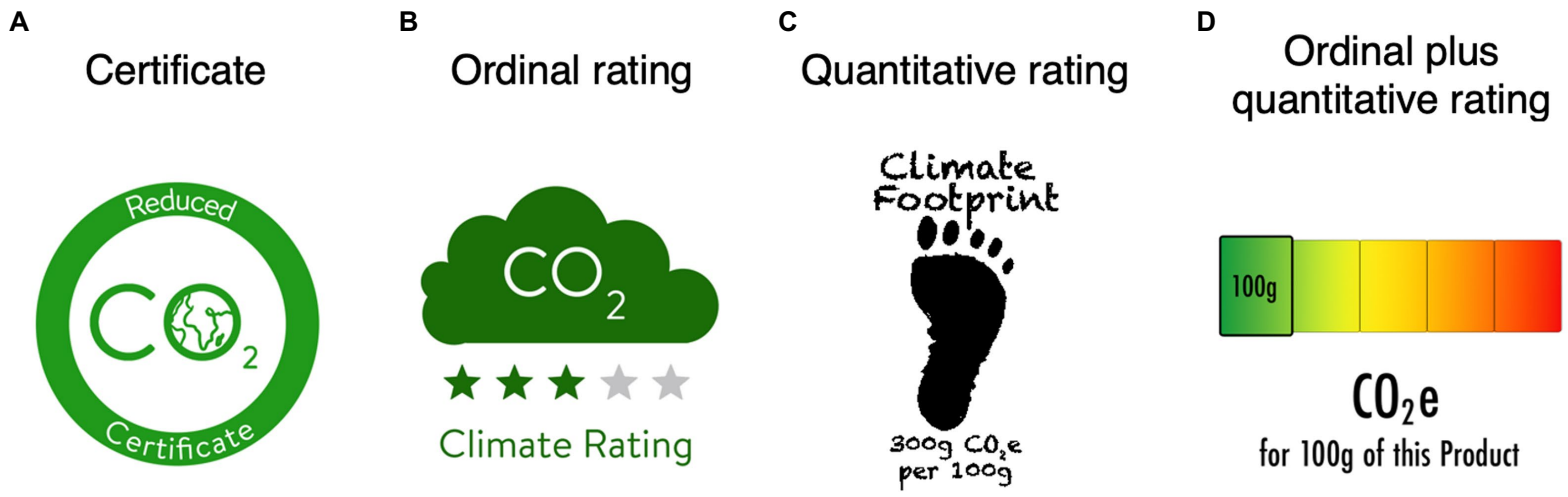
*Existing carbon footprint labels. Note*. Examples of the four label types: **(A)** a certificate label, **(B)** an ordinal rating carbon label, **(C)** a quantitative carbon label, and **(D)** an ordinal plus quantitative rating carbon label.

Certificate labels show consumers that either the product’s carbon footprint has been offset by the company that manufactured it, that the company that manufactured the product has stated that it will reduce its carbon footprint, or that the carbon footprint of that company’s product is less than that of a comparable product (those are the most common variants of certificate labels). Hence, such labels can only be attached to certified products. One such certificate label is the *Climatop label* (see Myclimate, 2022, displayed in Figure 1A) which was introduced in Switzerland in 2008 (similar labels are used in Thailand or the United States; see Liu et al., 2016). The Climatop label shows that the certified product, for example, a particular cream, has a lower footprint than comparable creams. Unlike most certificate labels (e.g., Climatop), *ordinal rating labels* could be attached to all products because they show a product’s overall carbon footprint by using, for example, a *star rating system* (e.g., from 0 stars = high emissions to 5 stars = low emissions; similar to a hotel rating) or a color-coding system (e.g., green = low emissions, orange = medium emissions, red = high emissions; so called Traffic Light Labels). One such ordinal rating label using a star rating system is the *M-Check* (see Figure 1B) which was introduced by Switzerland’s largest grocery retailer the Migros cooperation in 2021 (see Migros, 2022). *Quantitative labels,* unlike ordinal rating labels, show a product’s carbon footprint not by its membership in a particular ordinal category (e.g., 3 stars or orange), but by its effective CO_2_e emissions in g (e.g., 330 g; see Figure 1C). One such quantitative label is the Carbon Trust label, which was introduced in the United Kingdom. Similar labels exist in Taiwan, South Korea, and Japan (see Liu et al., 2016). What is unique about the Japanese label is that it also includes a pie chart showing where (production, distribution, etc.) the emissions come from in percentage terms (see EcoLeaf, 2022). The *ordinal plus quantitative rating* label, the last type of label, combines the ordinal rating label and the quantitative label. It shows not only the ordinal category in which the product’s emissions fall, but also the exact CO_2_e emissions of the product. One such *ordinal plus quantitative rating* label is the “indice carbone” of the French supermarket chain Casino (see Figure 1D).

### 2.2. Behavioral effects of carbon labels

Despite the large number and wide variety of existing labels, only a few studies examine their effectiveness in guiding consumers towards lower carbon food choices, particularly in the area of *grocery shopping*. One of these is the study by Vanclay et al. (2011), which examined whether the last type of label (i.e., the *ordinal plus quantitative rating label*), a so-called Traffic Light Label (TLL), attached to 37 products with high turnover in a supermarket, influenced their purchase. The TLL color (green, yellow, *black*) of a product was determined by comparing the carbon footprint of that product (e.g., a specific butter) to the average carbon footprint of other products in the same category (e.g., other butters). For example, if the product’s carbon footprint was below this average, it received a green label. Whether this so-called *relative color coding* is the best method is unclear. Nevertheless, the authors found a substantial, but non-significant *decrease* of sales (from 32 to 26%) of high carbon footprint (black labeled) products and a non-significant increase of sales (from 53 to 57%) of green labeled products. However, when the labeled products were also the *cheapest* (e.g., in the case of butter), a significant 20% increase in the purchase of green labeled products was observed. Although the effects in this study were small, the labels did appear to have an effect. Some more studies testing the impact of carbon labels on food choices are found in the *restaurant* area. Here too, carbon labels lead people to choose more environmentally friendly menus (see Brunner et al., 2018, or Visschers and Siegrist, 2015). But again, the observed effects were small (around 4 to 8%), and Spaargaren et al. (2013) only found an effect when labels were colored and supplemented with other information (e.g., posters) about the carbon impacts of food.

The question arises why is the impact of carbon labeling rather small? If you have to choose between *a kiwi and a pineapple*, environmental friendliness is only one of the many specifications to consider, along with price, for example. Accordingly, using a discrete choice experiment, Thøgersen and Nielsen (2016) showed that consumers’ choice, in this case for ground coffee, was more influenced by the price of the product and the presence of an organic label than by a *carbon label*. Nevertheless, the carbon label had a positive impact on choice (see also Hartikainen et al., 2014; Hieke et al., 2015; Feucht and Zander, 2017; Dihr et al., 2021, or Meyerding et al., 2019). Additionally, Thøgersen and Nielsen (2016) showed that their *colored* version of the label (i.e., an ordinal plus quantitative rating label) performed better than the one without colors, which accordingly only showed the CO_2_e content in g (i.e., a quantitative label). A similar result was observed in the study by Meyerding et al. (2019), who also used a discrete choice experiment to examine how 6 different labels affected tomato choices. Similarly, they found not only that colored labels were more effective than labels without color, but additionally that a simple TLL was as effective or more effective than more complex labels.

In summary, although research has shown that carbon labels can influence purchase decisions see also the reviews of carbon labels by Potter et al. (2021), Rondoni and Grasso (2021), Taufique et al. (2022) there is, in the words of Thøgersen and Nielsen (2016), “a lack of research on how to increase the effectiveness of such a label by optimizing its design” (p. 87).

## 3. Improvement of carbon labels

### 3.1. Relevant design elements

However, what could such *design elements* be? Typically, consumers spend only a few seconds on food choices. For example, 5 s for a milk decision in a realistic scenario, a grocery store, (see Thøgersen et al., 2012) or even only 500 ms for a binary decision of the preferred product from two products displayed on a computer screen (see Mormann et al., 2011). The speed of decision-making confirms that consumers’ food choices are not based on slow-moving, complex calculations, that require cognitive resources, but on the application of fast-moving, simple heuristics and cues (see Thøgersen et al., 2012; or Scheibehenne et al., 2007), primarily like the price rather than the carbon footprint of the products (Thøgersen and Nielsen, 2016; Meyerding et al., 2019; Carbon Trust, 2020). Accordingly, the eye-tracking study by Beattie et al. (2010) showed that consumers did not look at the carbon label first in 93% of the cases. Hence, they concluded that carbon labels must first and foremost become more *salient*.

Grocery shopping corresponds to a *visual search* for a specific target object (e.g., kiwi) in a scene cluttered with a variable number of other distracting objects (other fruits). It has been shown in laboratory experiments that *color* is the object’s feature that can most efficiently guide attention to the target object (see Wolfe and Horowitz, 2004). In this respect, the colors red, yellow, and green perform better than the colors blue and purple (see Lindsey et al., 2010). In addition, *colors* seem to have an effect on people’s *emotions*. For example, red seems to be associated with *negative* words (e.g., worse), whereas green is associated with *positive* words (e.g., best, see Moller et al., 2009; Pravossoudovitch et al., 2014). This makes perfect sense, because red is used in everyday life to *signal danger*, for example in warnings or stop signals. In addition, natural hazards such as fires are also red. Green, on the other hand, is used to signify something *positive*, such as safety, or go signals. In addition, growth in nature is green (see Moller et al., 2009). In a qualitative study by Carrero et al. (2021) where participants were asked about the emotions evoked by red and green carbon labels, said associations were confirmed by the participants. However, for grey/black colored labels e.g., the black Carbon Trust label or the TLL using black instead of red by Vanclay et al. (2011) participants’ associations were ambiguous (see also Pravossoudovitch et al., 2014, about reaction time and grey colored words). Accordingly, carbon labels should use colors or, more precisely, traffic-light colors (red, orange/yellow, green) because colors not only attract consumers’ attention, but also convey a message (see Thøgersen and Nielsen, 2016).

Accordingly, studies in the *healthy food* domain have already shown that traffic light colors work not only better than other colors (e.g., purple, white, blue; Van Epps et al., 2021) but also, for example, as star ratings (e.g., Health Star Rating System; Egnell et al., 2020). Star ratings appear to influence food choices only when a corresponding color is added (e.g., green for 5 stars; Pettigrew et al., 2020). To our knowledge, such studies or findings do not yet exist with respect to carbon labels.

In addition, carbon labels should be *easy* to understand. As mentioned, humans do not like to make complicated calculations. This is also shown when people have to assess (calculate) the healthiness of a food by weighing and adding up the different nutritional information such as fat, sugar, etc. Furthermore, it has been shown that, for example, the weighing itself can be slightly changed depending on the context (see Reijnen et al., 2019). Accordingly, it has been found that so-called summary labels (indicating the final product of the calculation by, for example, a color), attached to the front of the package, help consumers to recognize healthier products (see Hagmann and Siegrist, 2020).

This simplification is also the idea behind carbon labels. However, consumers still find certain carbon labels too difficult to understand. For example, in the study by Carrero et al. (2021), consumers thought that the carbon labels tested showed (dangerous) ingredients. It is possible that the label design showing a cloud with the words “CO_2_” triggered such an association. Overall, consumers appear to find the carbon TLL with red to green colors easier to understand, more helpful in the decision-making process, and more popular than the original carbon trust label or a TLL with black rather than red for high carbon products (Sharp and Wheeler, 2013).

In summary, we suggest that labels should be designed according to the principles of Attractive, Easy and Timely. In other words, they should be Attractive by attracting attention and providing information in an appealing design. Easy by being easy to understand. This by using symbolic language such as colors and signs that are commonly known and make the products easy to compare. Finally, they should be Timely by displaying the information at the moment of decision. This can be achieved by placing labels on the front of the package, as eye-tracking studies have shown that consumers prefer to pay attention to information on the front of the package (e.g., health claims and labels) than on the back (e.g., nutritional information, see Bartels et al., 2018). These design principles are based on the EAST framework for behavioral interventions (see Hallsworth et al., 2014).

### 3.2. New types of carbon labels

**Figure 2:**
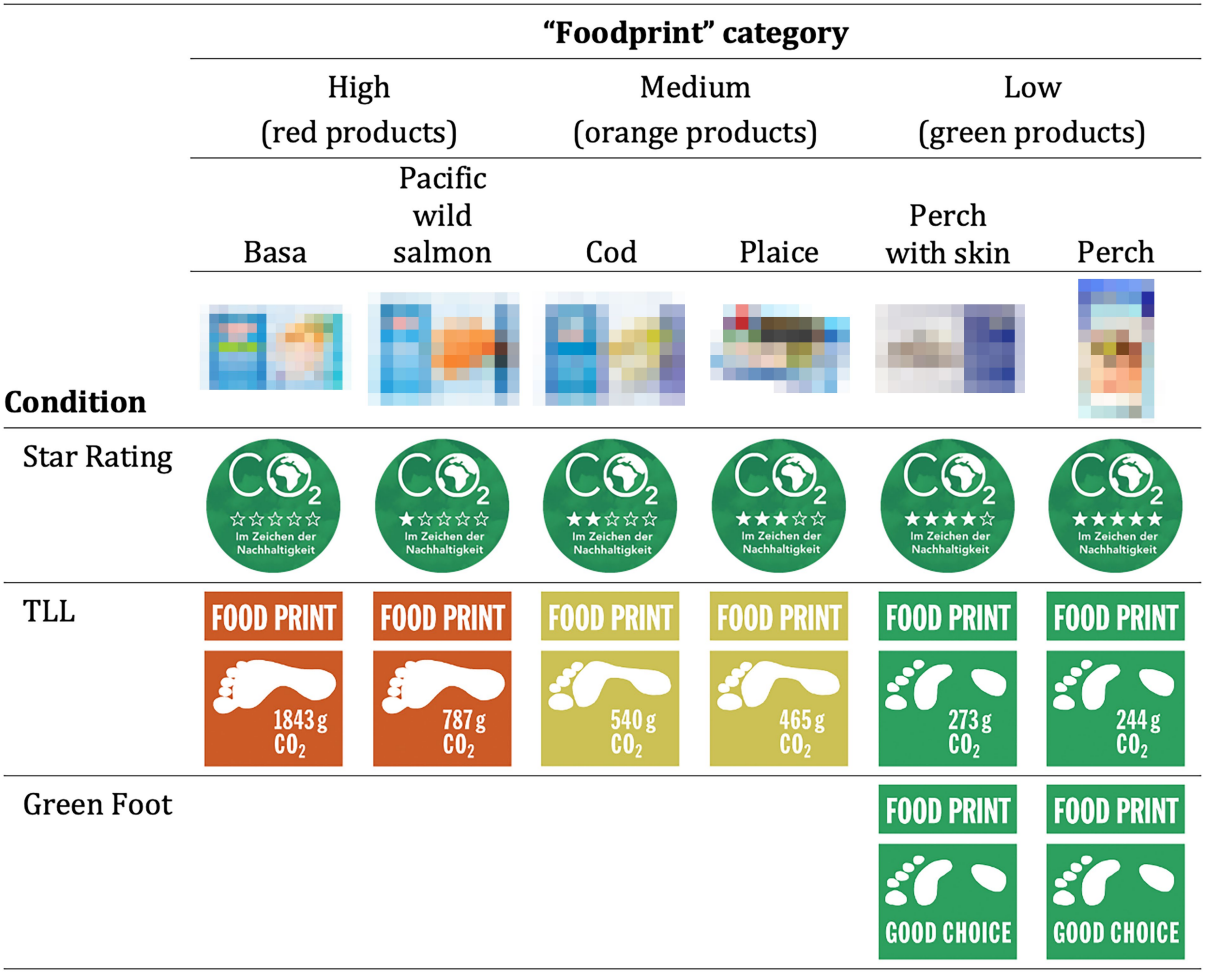
Example of the three different labels tested in the product category “fish.”

Based on these design principles, we designed 3 types of labels. The first type of label is the Star Rating Label (see Figure 2) like the M-Check label, which falls into the category of ordinal rating labels. The level of the product’s carbon footprint is shown by *0 to 5 stars*. The design was thereby based on the well-known hotel rating system, as well as the Energy Star label used for appliances in many countries. Also added were the words “CO_2_” (with the planet symbol in the O) and “in the name of sustainability” on a green background. Accordingly, we also used design elements from existing labels, such as the Climatop label. The second type of label is the TLL (see Figure 2), in which the level of the product’s footprint is shown by either a green (low emissions), an orange (medium emissions), or red color (high emissions; see Vanclay et al., 2011; Thøgersen and Nielsen, 2016; Meyerding et al., 2019). This label was designed in collaboration with two designers from the Zurich University of Applied Arts. Contrary to other labels, it was not the foot itself that was colored, but the area around the foot. This allowed more area of the label to be inked, which should enhance the color effects described above. Also, depending on the color of the label, the footprint has a slightly different shape (e.g., green = a lighter print). Furthermore, the words “Carbon Foodprint” and the numerical amount of CO_2_e were added. The label thereby falls in the category ordinal plus quantitative rating label. The third type of label is the “Green Foot” label, which falls into the category certificate labels. The design is similar to that of the green TLL, but without the quantitative information on CO_2_e emissions. The low emission is shown by the addition “Good Choice.” Similar certificate labels are the Climatop or versions of the Carbon Trust label (see Supplementary material 4 for a comparative tabular overview of the labels).

The aim of the study is to answer the question of whether carbon emissions from shopping can be reduced by carbon labels, respectively which newly developed type of label is most effective in this regard. To test this, we designed an online study in which participants were randomly assigned to one of three labeling conditions (type of label: star rating, TLL, green foot) or a control condition with no labeling. Regardless of the condition, participants were presented with 20 different food categories (e.g., pasta, vegetables). Each category contained 6 products - depending on the condition with a specific type of label or not - from which participants had to choose 1 product by putting it in the shopping cart. Thereby we measured, the total carbon emissions of the shopping cart, the average rating of the products chosen in each food category and the number of red, orange and green products chosen. Furthermore, it was investigated which type of labeling is most accepted and which institution should initiate such a labeling regarding its trustworthiness.

## 4. Materials and methods

### 4.1. Participants

Four hundred two participants aged 19 to 65 years old (*M*_age_ = 26.05; *SD_age_* = 6.45; 61.7% female) from ZHAW Zurich University of Applied Sciences and the greater area of Zurich took part in this computer-based online study. As an incentive, participants could enter a raffle for one of two iPads (which a total of 79.4% did) or if a student of the ZHAW School of Applied Psychology receive course credit instead (which 6.2% overall did). All participants gave informed consent.

### 4.2. Stimulus material

As stimulus material served images of 120 food (and beverage) products from 20 different categories: milk, cheese, citrus fruits, fish, coffee, pasta, rice, sausages, meat, etc. Foods were selected to cover the full range of everyday needs. The images (taken with permission from an online store of one of the largest Swiss retailers) were supplemented with the name, price, quantity as well as several CO_2_e relevant specifications of the product (see Figure 2). More precisely, these specifications were: Country of origin (e.g., Switzerland), transport mode (ground or by air), preservation mode (e.g., fresh or dried) and cultivation (organic or conventional) of the product (see eaternity^2^ calculator for details). This information was provided to rule out the possibility that the effect of the carbon labels - compared to the control group–was due only to the lack of additional information to calculate CO_2_e levels. Depending on which of the four conditions participants were randomly assigned to, they saw either, a grocery shop where none of the products were supplemented with a label (control group), only some products were supplemented with a label (see Green Foot condition), or all products were supplemented with a label (Star Rating and TLL conditions). Since the labels have already been described in detail in the introduction, there are just 2 additions. First, the total number of CO_2_e was only shown in the TLL condition, since neither “carbon reduced” labels (e.g., Climatop) nor star rating labels (e.g., Migros M-Check) normally show these numbers. Second, the rating or color coding is relative, that is, per product category (see fish example of Figure 2; according to Vanclay et al. (2011)) and not across all the products.

### 4.3. Procedure

**Figure 3:**
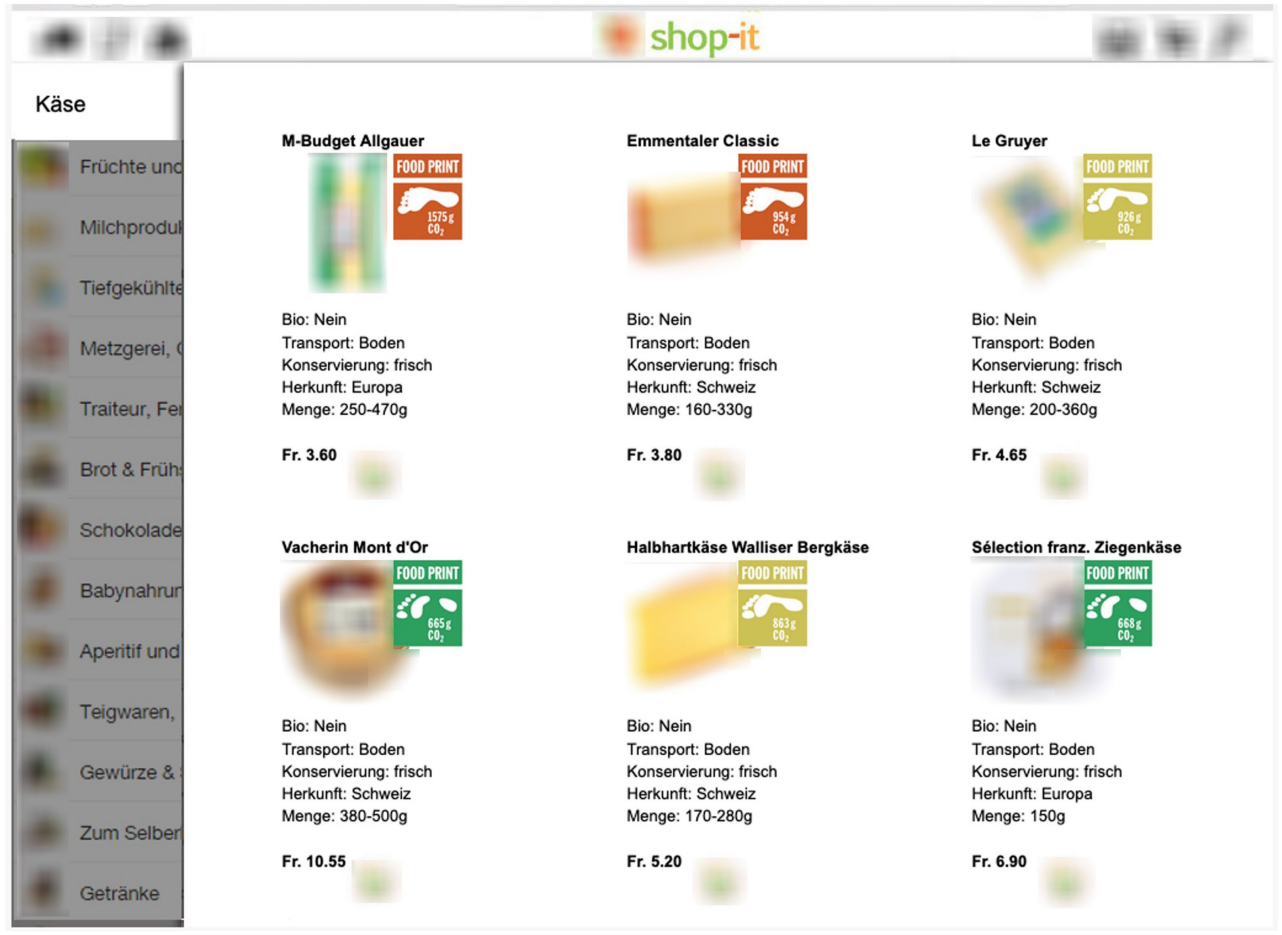
Stimulus material. Examples of the choice task in the TLL condition.

At the beginning of the study participants had to imagine that they had to buy 20 food products for a housewarming party with friends from a new online shop (here called shop-it). In doing so, they were reminded that as students, they have a limited budget. Note that nothing was said about sustainability, labels, etc. Participants were then guided through the shop or products by being shown 20 pages (see Figure 3), each containing 6 food products of a particular category (e.g., 6 different cheeses). This was to ensure that all participants saw the same products. Participants could then buy^3^ a product by clicking on the shopping basket symbol below each product. The order of the categories was kept constant in all conditions but the position of the products per page was randomized.

After the food shopping task, participants had to answer a series of questions. For example, in the label conditions, there were questions about the acceptance of the corresponding label such as “I consider the CO₂e label to be credible” (on 5-point Likert scales, from-2 “strongly disagree” to 2 “strongly agree”). In addition, all participants had to answer the question as to how trustworthy^4^ they would rate labels certified by various institutions such as private companies or the European Union. Trustworthiness in a label is an important factor for its acceptance and previous studies showed that consumers have rather low trust in carbon labels (see Feucht and Zander, 2017). We also included a control question (i.e., an item “Please tick the answer ‘Agree’ for control purposes”) to check the validity of participants’ responses. The carbon label questions were taken from Weinrich and Spiller (2016; with their approval) and adapted to our context on carbon footprint labels. Since these questions are not relevant with respect to the aim of the study, we do not address them further. At the end of the study, we assessed participants’ demographic data (e.g., age, sex).

Regarding the food shopping task, we first calculate the CO_2_e (in g) emissions of the products purchased, that is, of those products that ended up in the shopping basket. The CO_2_e of a product was thereby calculated per 100 g. Second, we rated the products within each food category by the number of stars they would have received under the Star Rating label condition (0 = high emission, to 5 = low emission). This scale made it possible to assess if labels affect choices differently in each of the categories. This way of rating makes comparisons easier as the different categories have large differences in the mean and range of their carbon footprint (for example meat from 486 g to 7,287 g CO_2_e, but citrus fruits only from 24 g to 61 g CO_2_e). Third, we calculated the number of products purchased within the high (red), medium (orange), and low (green) footprint categories.

## 5. Results

### 5.1. Participants excluded

From the 449 participants that completed the study, 33 participants (7.3%) who needed less than 10 or more than 60 min to complete the study were excluded from the analysis. Furthermore, 14 participants (3.1%) who did not answer the control question correctly were excluded.

### 5.2. Shopping task

#### 5.2.1. Overall CO_2_e emissions (in g)

**Figure 4:**
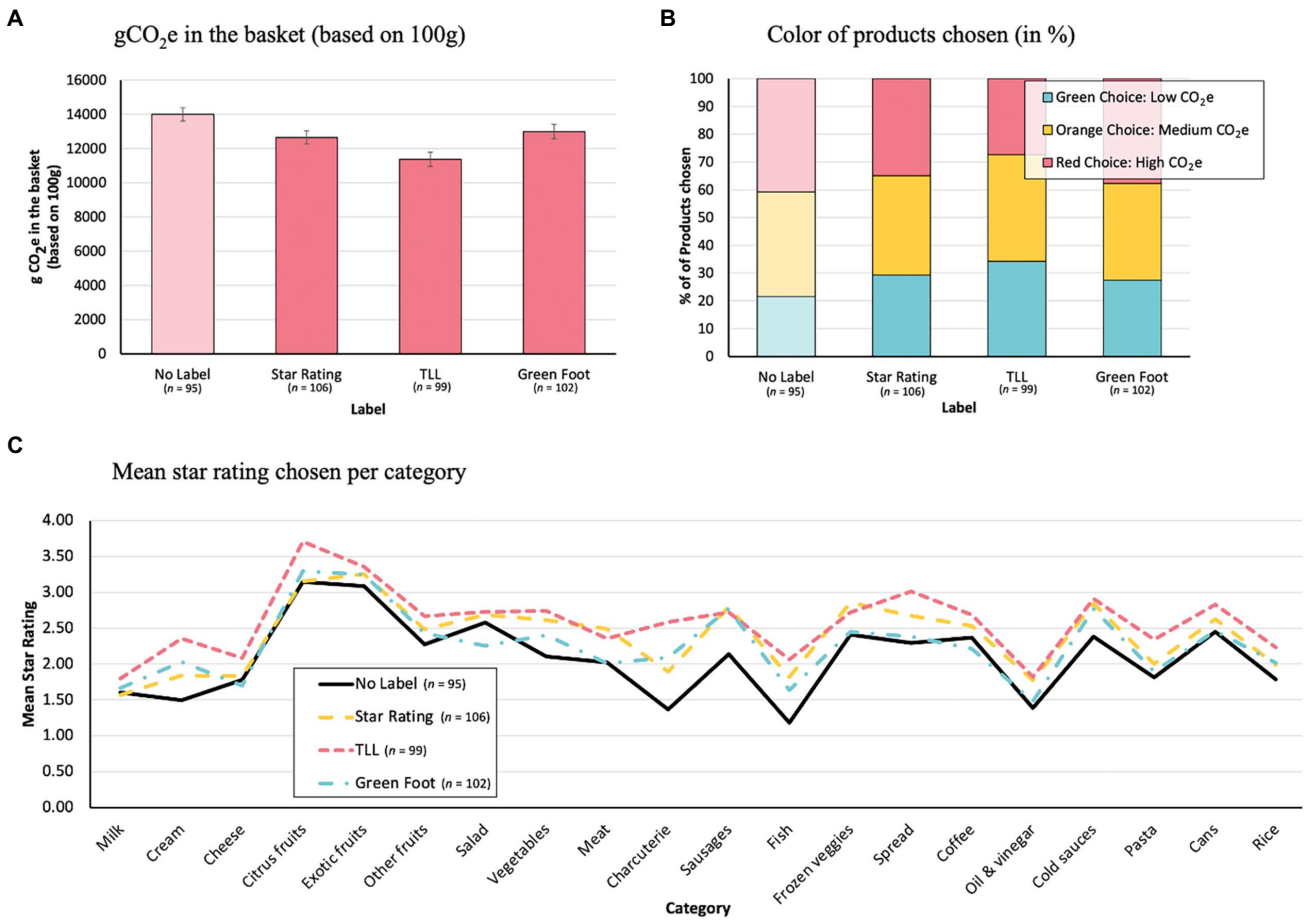
Results of the different outcome measures: **(A)** gCO_2_e of the products (based on 100g) in the shopping basket, **(B)** color of the product chosen in %, **(C)** mean star rating of the products chosen in each category.

A *planned contrast* (under a one-way ANOVA) showed that the total g CO_2_e of the products bought in the no label (control) condition was significantly higher than in the label conditions (Star Rating, TLL, Green Foot), *t*(398) = 3.76, *p* < 0.001, *d* = 0.44. Tukey adjusted post-hoc tests showed a significant difference between the control condition and: the Star Rating label condition, *t*(398) = 2.73, *p* < 0.05, and the TLL condition, *t*(398) = 4.75, *p* < 0.001, but not the Green Foot label condition, *t*(398) = 1.81, *p* = 0.27. Within the label conditions, there was only a significant difference between the TLL condition and the Green Foot Label condition, *t*(398) = 3.01, *p* < 0.05 (all other comparisons were not significant: *t* < 2.13, *p* > 0.14; see Table 1 for the specific values). Overall, this suggests that the TLL is the most beneficial and the Green Foot label the least beneficial label in terms of total g CO_2_e reduction (see Figure 4A).

**Table 1:** Outcome measures for the shopping task per condition.

Measure		Control (*N* = 95)	Star rating (*N* = 106)	TLL (*N* = 99)	Green foot (*N* = 102)
CO_2_e (in *g*)		13′992.7^a^ (3′740.0)	12′645.4^b,c^ (3′984.3)	11′366.2^b^ (4′069.6)	12′985.2^a,c^ (4′297.6)
Footprint (in number of products bought)	Green	4.32^a^ (1.97)	5.86^b^ (2.85)	6.84^c^ (3.18)	5.47^b^ (2.54)
	Orange	7.52^a^ (2.18)	7.14^a^ (2.27)	7.69^a^ (2.53)	6.98^a^ (2.11)
	Red	8.17^a^ (2.70)	7.00^b^ (3.25)	5.47^c^ (3.37)	7.55^a,b^ (3.15)

Standard deviations are given in parentheses. Letters indicate Tukey-adjusted comparisons; same letters indicate that no significant difference between conditions exists.

#### 5.2.2. Label effects per category (measured with the carbon star rating)

A repeated measure ANOVA showed a significant main effect for condition (labels and no label), *F*(3, 398) = 14.15, *p* < 0.001, *η^2^* = 0.10, as well as a category, *F*(19, 7,562) = 41.51, *p* < 0.001, *η^2^* = 0.09, but no significant Condition × Category interaction, *F*(57, 7,562) = 1.07, *p* = 0.33. Tukey adjusted post-hoc tests showed again a significant difference between the control condition and: the Star Rating label condition, *t*(398) = 3.86, *p* < 0.001, and the TLL condition, *t*(398) = 6.30, *p* < 0.001, but not the Green Foot label condition, *t*(398) = 2.23, *p* = 0.16. Within the label conditions, there was only a significant difference between the TLL condition and the Green Foot Label condition, *t*(398) = 4.16, *p* < 0.001 (all other comparisons were not significant: *t* < 2.57, *p* > 0.06). These results are thereby in line with the results of overall CO_2_e emission (in g) calculations and show that labels have an effect on choice. Though the labels had no differential effect per category (no interaction), there are differences between the categories (see Figure 4C). However, this seems to be more due to differences in preference of certain products. Thus, participants in the control condition already preferred products with low carbon emissions in some food categories (e.g., citrus) and products with high emissions in other categories (e.g., fish). Nevertheless, the results suggest that labels can have a positive effect regardless of category.

#### 5.2.3. Number of products purchased per footprint category

Do labels affect product purchases (compared to the control group) differently in the red (high), orange (medium), or green (low) footprint categories, and if so, how (see also Table 1)? To investigate this, we conducted a multinominal regression analysis with Tukey adjusted contrasts.

The calculated regression was significant, *χ*^2^ (6) = 115.75, *p* < 0.001, *R*^2^ = 0.02, indicating a differential label effect regarding the purchase of green, orange, and red products. The subsequently calculated planned contrasts, for example, showed that all label conditions led participants to purchase *more green* products than in the control condition (Star Rating label condition: *t*(8) = 5.44, *p* < 0.01; TLL condition: *t*(8) = 8.91, *p* < 0.001; Green Foot label condition: *t*(8) = 4.58, *p* < 0.01). Within the label conditions regarding green choices, there was a significant difference between the TLL condition and Green Foot condition, *t*(8) = 4.54, *p* < 0.01, and the TLL condition and the Star Rating label condition, *t*(8) = 3.65, *p* < 0.05, but not between Star Rating condition and Green Foot label condition, *t*(8) = 0.84, *p* = 0.83. However, in the *orange* category, there were no differences in products purchased between the label conditions and the control condition, or between the label conditions themselves (all *t* < 2.29, *p* > 0.17). Finally, the Star Rating label condition, *t*(8) = −3.75, *p* < 0.05, and the TLL condition, *t*(8) = −8.79, *p* < 0.001, but not the Green Foot label condition, *t*(8) = −2.20, *p* = 0.21, made participants buy *less red* products compared to the control condition. As regards the green products, differences between the labels were significant between the TLL condition and the Star Rating condition, *t*(8) = 5.26, *p* < 0.01, and the TLL condition and the Green Foot label condition, *t*(8) = 6.77, *p* < 0.001, but not between the Star Rating condition and the Green Foot label condition, *t*(8) = 1.57, *p* = 0.44. Accordingly, the labels, especially the TLL, had a positive effect in the green (more purchases) and in the red (less purchases) footprint category (see Figure 4B). However, no significant effects could be found for any of the labels in the orange footprint category.

### 5.3. Questions

#### 5.3.1. Label acceptance

The overall consistency of the questions was good (α = 0.86, item loadings from 0.46 to 0.80). A calculated one-way ANOVA showed a significant main effect for condition (note, only the three label groups saw these questions), *F*(2, 304) = 5.06, *p* < 0.01, *η*^2^ = 0.032. Tukey adjusted post-hoc tests showed significant differences between the Star Rating label (*M* = −0.06, *SD* = 0.76) and the TLL (*M* = 0.22, *SD* = 0.71), *t*(304) = 2.83, *p* < 0.05, and the TLL and the Green Foot label (*M* = −0.05, *SD* = 0.64), *t*(304) = 2.70, *p* < 0.05, but not between the Star Rating and the Green Foot label, *t*(304) = 0.11, *p* = 0.99. Overall, participants’ label acceptance was highest for the TLL, although the effect is rather small. The translated questions and means are shown in Supplementary Table 1.

#### 5.3.2. Controlling institution

There was a significant effect regarding trustworthiness of the controlling institution, *F*(5, 1,530) = 127.61, *p* < 0.01, *η*^2^ = 0.29. Participants regarded a carbon label awarded by the State (i.e., Switzerland, *M* = 0.84, *SD* = 0.67) and the WWF (*M* = 0.71, *SD* = 1.04) as most trustworthy. Tukey-adjusted post-hoc tests found no difference between these two institutions, *t*(306) = 2.50, *p* = 0.13. Comparably (i.e., State and WWF), lower values of trustworthiness could be found for the federation of Swiss food industry (*M* = 0.31, *SD* = 1.04), the EU (*M* = 0.25, *SD* = 0.93), private companies (*M* = −0.06, *SD* = 1.02) and international corporations (*M* = −0.51, *SD* = 1.02; all *t* < 5.62, *p* < 0.001). For a graphical representation see Supplementary Figure 1.

## 6. Discussion

Overall, our results show that *carbon labels* make consumers choose more environmentally friendly food products, especially under the *TLL*, an *ordinal plus quantitative rating* label. The TLL resulted in the largest reduction in *overall CO_2_e emissions* (in g), shown by the purchase of more *green* (e.g., low carbon footprint meat such as chicken) and less *red* (e.g., high carbon footprint meat such as beef) products. Thus, these results are consistent with those of existing research (see Vanclay et al., 2011; Thøgersen and Nielsen, 2016; Meyerding et al., 2019). The other labels, the *Green Foot* (a certificate label) and also the *Star Rating* label (an ordinal rating label) showed similar, albeit weaker, effects. Finally, TLL affected choice in all food categories and was also the most widely accepted by participants. Unlike other studies, in this study, not only was the effectiveness of multiple carbon labels (3 labels) tested simultaneously, but they were also tested among a broader range of products instead of just one, such as coffee or tomatoes (Thøgersen and Nielsen, 2016; Meyerding et al., 2019, respectively), or a few products [four categories in Vanclay et al. (2011)] and in a new setting, a realistic online shopping scenario.

However, despite the proven effectiveness of carbon labels, there are still some critical aspects in this regard that need further investigation:

### 6.1. Reference frame for reporting carbon emissions

For example, our labels show CO_2_e emissions in grams *per 100 g* (or 100 ml) of a product. Alternatively, you could do this per *serving size* or *package size*. For example, 100 ml of wine, which is also considered a serving size in Switzerland, has about 360 g CO_2_e, whereas 100 ml of beer has only 115 g CO_2_e (Saxe, 2010). However, a standard size of beer is usually 250 ml, which then leads to the higher CO_2_e footprint of 287 g CO_2_e, which is then comparable with wine. Which measure is better is still up for debate. Regarding healthy food labels, the 100 g comparison is nowadays the most widely used (e.g., Nutri-Score, TLL, or the Chilean warning label). This is because specifying by portion size has the disadvantage that non-standard portion sizes can easily be reduced by the retailer to make the values turn out better. This could also happen with carbon labels by offering, for example, smaller package sizes. People would then probably buy several small packages, which is no better than buying one large package. Out of curiosity, we ran our analysis again, taking package sizes into account. For example, if a participant chose the 150 g goat cheese, the value of 1,002 g CO_2_e instead of 668 g CO_2_e was newly included in the calculation. The label effects found were similar, indicating that the effect of carbon labels per 100 g is maintained even when differences in package size are considered (see Supplementary material 3 for more information).

### 6.2. Reference frame for color coding

Similar to Vanclay et al. (2011), and also Muller et al. (2019), the color of a product’s carbon label was determined relative to the average emissions of the other products in the same category (i.e., relative color coding). This approach is based on the idea that consumers usually choose between products (e.g., different types of cheese) of the same category when making food decisions and the label should accordingly guide them to, for example, the cheese with the lowest carbon footprint. This seems feasible as a color coding across all products as for example the logo of Raisio (2022) would make it difficult to select an environmentally friendly cheese, for example, as most of them would get the color red. The downside, however, is that consumers could be left with the impression that a green product in one category (e.g., meat) is just as carbon friendly as a green product in another category (e.g., fish). This could then lead to, for example, increased purchase of green-labeled products in a high-carbon category such as meat. Note that this has not yet been documented for relative color coding, so these assumptions are so far only speculative. Although, the results of Brunner et al.’s (2018) study on menu choice suggest that may even cross-product color coding could lead to such effects.^5^

### 6.3. Aspects included in the calculation of the label

Most carbon labels – like ours – do not consider the impacts on, for example, biodiversity or water use (see the eaternity label^6^, for a label that does). Whether the consideration of these further characteristics has an additional benefit is still under discussion (little benefit has been reported when characteristics strongly correlate with each other, see Muller et al., 2019). It is also unclear whether it is better to report a separate value for each characteristic (see eaternity label) or a total value averaged over all characteristics [as suggested by Foundation Earth (2022)]. The total value, however, offers the possibility of manipulation, since a bad value on one characteristic can be compensated with a good value on another characteristic. This is what happened with the Nutri-Score health label, where Nestlé, for example, changed the recipe of its Nesquik cereals to give them a Nutri-Score score of A (instead of C, see Nestlé, 2021). However, since the sugar content is still very high (22 g per 100 g), it cannot be considered a healthy cereal. On the other hand, if you have to integrate the values of several characteristics into a single total value, you can easily be misled. This was found not only for healthy food labels (see Reijnen et al., 2019), but also in the study by Meyerding et al. (2019), where products with a lower overall carbon footprint but orange, green, and red ratings for various CO_2_e-related attributes influenced purchase decisions less than a label with a higher overall footprint.^7^

*Negative labeling*. In contrast to all the approaches mentioned so far, one could think about labeling only those products that have a high carbon footprint or even poor scores on other attributes such as biodiversity. Although there are no studies to date examining the impact of red/black traffic light labels attached only to products with high carbon footprints, there is evidence that negative labels may influence purchasing decisions more than positive ones (Grankvist et al., 2004; Meyerding et al., 2019).^8^ For example, Van Dam and De Jonge (2015) found that a non-organic label applied to conventionally produced products led to a higher preference for organic food than an organic label applied to organic food. The larger effect for negative labels can be explained by loss aversion, in which people weigh the risk of a negative outcome more heavily than that of a positive one (Kahneman and Tversky, 1979). The results of our study do not show a superior effect of negative labeling, only that TLL overall are more effective than labeling just green products with a certificate label.

In terms of limitations, one could cite the usual concerns about online studies (i.e., external validity) and the appropriateness of the sample (i.e., students). Another limitation could be that we did not record whether participants understood the meaning of the different terms. For example, Hartikainen et al. (2014) found that participants were unaware that labels assess CO_2_e emissions. Perhaps carbon labels influence choice even if consumers do not really understand the concept behind the label, but as the study by Li et al. (2017) shows, carbon labels affect consumers more when they understand it. We therefore propose to complement the simple TLL with a QR code that directs to a website or app where additional information about the product and the calculation of the carbon label can be found (this analogous to websites such as^9^ or^10^). Thereby the carbon label and the websites should be administrated by an organization such as the WWF or the state, as they are perceived as most trustworthy in our study (see also Thøgersen and Nielsen, 2016) and trustworthiness seems to affect the effectiveness of carbon labels (see Li et al., 2017).

Overall, this study adds to the existing carbon label literature by testing in a shopping task, three different label formats, whereby two have to our knowledge not been empirically tested so far (carbon certificate label and rating label without color). The study underpins the findings of other authors that carbon TLLs are more accepted and more effective than other labels tested so far. We also identified research gaps, such as a lack of research about the influence of the reference frame on TLL’s effectiveness, or a lack of research about negative carbon labels. Nevertheless, well designed carbon labels can influence decision making and lead consumers to carbon friendlier food choices.

## Data availability statement

The datasets presented in this article are not readily available because only aggregated data are shared. Requests to access the datasets should be directed to swen.kuehne@zhaw.ch.

## Ethics statement

Ethical review and approval was not required for the study on human participants in accordance with the local legislation and institutional requirements. The patients/participants provided their written informed consent to participate in this study.

## Author contributions

SK and ER: made an equal substantial, direct, and intellectual contribution in all stages of the work. LLV and MB: were involved in the design and programming of the study. All authors contributed to the article and approved the submitted version.

## Funding

Open access funding provided by Zurich University of Applied Sciences (ZHAW).

## Conflict of interest

The authors declare that the research was conducted in the absence of any commercial or financial relationships that could be construed as a potential conflict of interest.

## Publisher’s note

All claims expressed in this article are solely those of the authors and do not necessarily represent those of their affiliated organizations, or those of the publisher, the editors and the reviewers. Any product that may be evaluated in this article, or claim that may be made by its manufacturer, is not guaranteed or endorsed by the publisher.

## Footnotes

^1^ https://www.ecolabelindex.com/ecolabels/

^2^Unfortunately, the eaternity calculator is not publicly available anymore. The data of the life cycle assessments are based on evaluations of different Universities and other research institutes, see: www.eaternity.org

^3^Note, the buying decision was hypothetical. Recent research shows that a hypothetical bias is less likely when it comes to environmental attributes of food. For example, Aoki and Akai (2022) did not find a hypothetical bias in the WTP for carbon emissions in food.

^4^A label is considered “trustworthy” if people judge the information (e.g., a claim) provided by the label to be credible. For this assessment, consumers consider cues such as which organization is behind the label (Gorton et al., 2021). If trustworthiness is present, it seems to moderate the impact of a label, that is, whether the consumer considers the label and decides accordingly (see Li et al., 2017). However, if a consumer perceives a label only as “green-washing,” it is perceived as less trustworthy (see Ye and Wildschut, 2009), hence making it less effective.

^5^In Brunner et al. (2018) study on days on which a green labeled meat dish was available, sales of this meat dish increased, and sales of the green labeled fish dish decreased, which resulted in a higher carbon footprint overall.

^6^ www.eaternity.org

^7^Note, in the study of Meyerding et al. (2019) a label with an orange aggregation footprint, a green overall and a red reduction footprint had a lower part-worth utility than a label where all footprints were orange, although the actual carbon footprint was lower for the first label.

^8^Note, Meyerding et al. (2019) found higher part-worth utilities in the CBCA for red then for green labels. Grankvist et al. (2004) found that participants with intermediate environmental concerns are especially affected by red labels, whereby participants with high environmental concerns are affected equally by green and red labels. People with low environmental concerns were not affected by the labels at all.

^9^ www.codecheck.info

^10^ www.openfoodfacts.org
