# Supplementary material for: Can carbon labels encourage green food choices?
Source: Front Psychol. 2023 Jan 27;13:902869. doi: 10.3389/fpsyg.2022.902869 (PMC9912457; doi:10.3389/fpsyg.2022.902869)
Supplement: Supplementary file 1 [file Table_1.DOCX]

Supplementary Material

# Descriptive statistics of label acceptance

| Items: Mean (SD) | **Star Rating**  (*N* = 106) | **TLL**  (*N* = 99) | **Green Foot**  (*N* = 102) |
| --- | --- | --- | --- |
| Overall | -0.06 (0.76) | 0.22 (0.71) | -0.05 (0.64) |
| I consider the CO₂ label to be credible. | 0.38 (0.91) | 0.52 (0.85) | 0.46 (0.89) |
| The CO₂ label makes it easier for me to compare products. | -0.06 (1.24) | 0.30 (1.09) | 0.07 (1.06) |
| Products with such a label catch my interest. | 0.16 (1.26) | 0.38 (1.28) | 0.17 (1.01) |
| I can imagine what is behind the CO₂ label. | 0.38 (0.98) | 0.71 (0.85) | 0.30 (0.99) |
| The CO₂ label gives me precise information without having to read much. | -0.17 (0.97) | 0.16 (1.11) | -0.31 (1.02) |
| The CO₂ label gives me the opportunity to know more about a product without having to inform myself in detail. | 0.14 (0.97) | 0.35 (1.03) | 0.04 (0.99) |
| I would notice products with this label in the supermarket. | 0.01 (1.17) | 0.55 (1.05) | 0.34 (0.93) |
| I would look for products with such a label in the supermarket. | -0.31 (1.17) | 0.01 (1.15) | -0.25 (1.02) |
| I would look for products with such a label at the weekly market. | -0.69 (1.21) | -0.52 (1.12) | -0.66 (1.07) |
| I would look for products with such a label at the butcher. | -0.43 (1.32) | -0.26 (1.29) | -0.65 (1.14) |

**Supplementary Table 1.** Outside the parentheses are the mean values, inside the parentheses are the standard deviations. Cronbach’s alpha: 0.86.

# Perceived trustworthiness of labels from different institutions

***
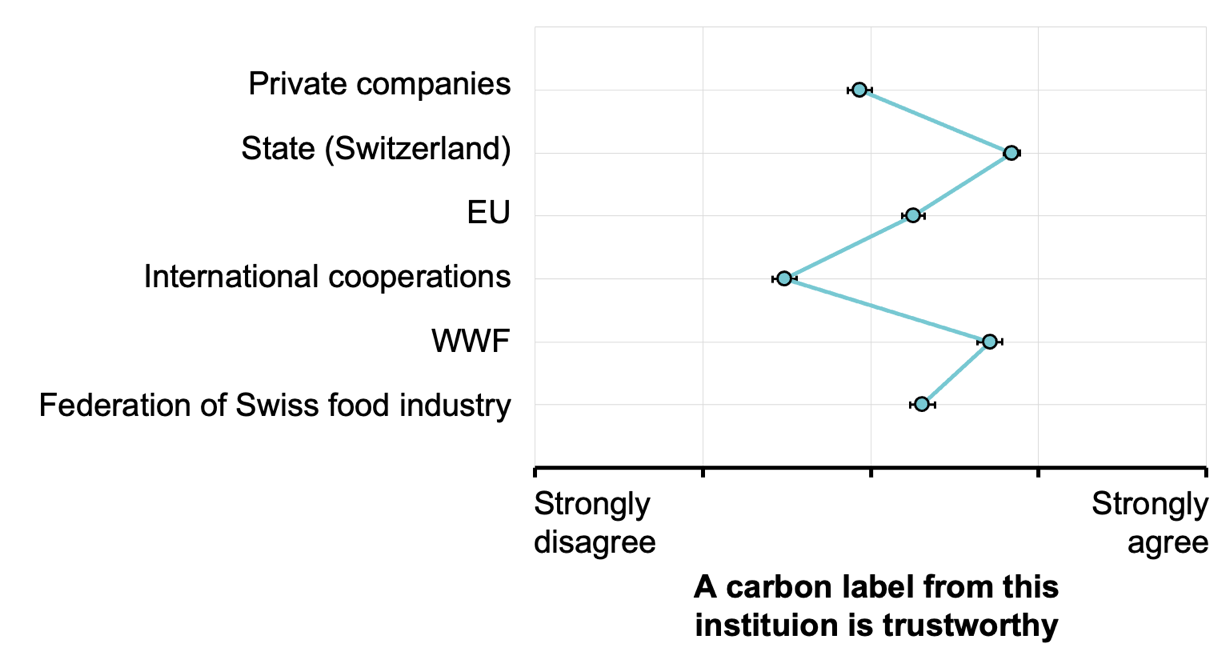
***

**Supplementary Figure 1.** Error bars represent standard errors*.*

# Total g CO_2_e in the shopping basket scaled on product size

A calculated ANOVA with a planned contrast between label vs. no label conditions over the aggregated purchase data revealed a significant effect, *t*(398) = 3.09, *p* < .01, *d* = 0.36; indicating that the carbon footprint was lower in the label conditions than in the control condition. Tukey adjusted post-hoc tests showed significant differences between the TLL and the control condition, *t*(398) = 4.34, *p* < .001, as well as between the TLL and the Star Rating label, *t*(398) = 2.72, *p* < .05, and between the TLL and the Green Foot label, *t*(398) = 2.85, *p* < .05. The post-hoc tests between the Star Rating label and the control condition, *t*(398) = 1.72, *p* = .31, and between the Green Foot label and the control condition, *t*(398) = 1.55, *p* = .41, and between the Star Rating label and the Green Foot label, *t*(398) = 0.17, *p* = 1.00, were not significant.


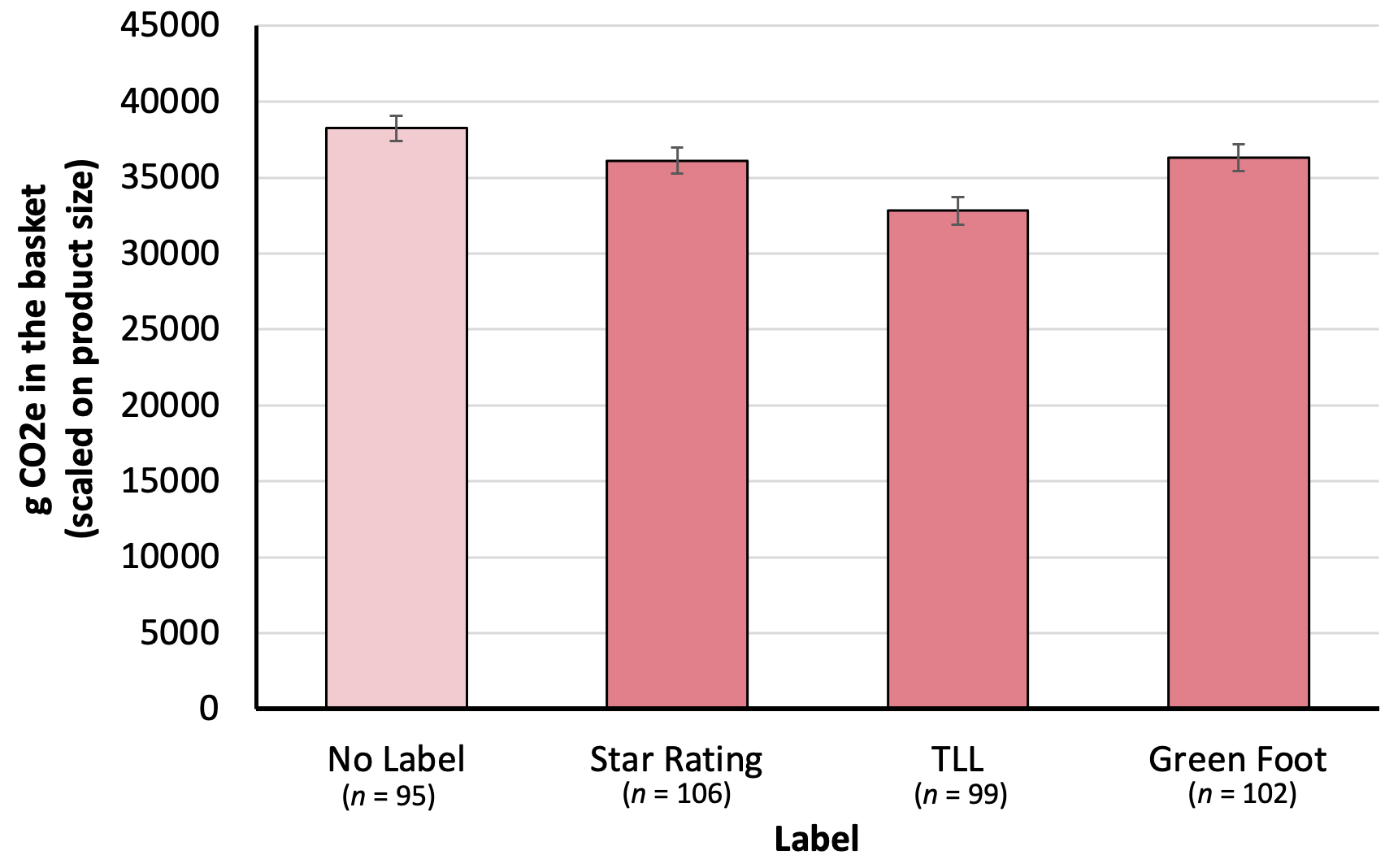


**Supplementary Figure 2.** Error bars indicate standard errors.

# Characteristics of the different types of labels

| Characteristics | **Star Rating**  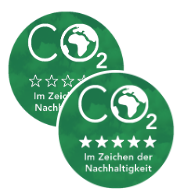 | **TLL**  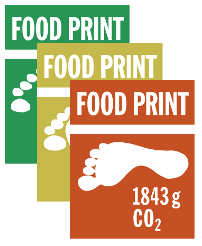 | **Green Foot**  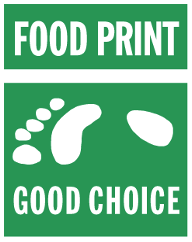 |
| --- | --- | --- | --- |
| Label type | Ordinal rating | Ordinal plus quantitative rating | Certificate |
| Label attachment | On all products | On all products | Only on the most carbon friendly products of each category |
| Rating system | Stars: 0 (high) to 5 (low emissions) | Color: red (high), yellow (medium), green (low emissions) | None |
| Quantitative CO_2_e value? | No | Yes | No |
| Symbolic language | Green color; globe | Different colors for differently shaped footprints (from light = green to strong = red) | Light, green colored, footprint |
| Text | “CO_2_” and “in the name of sustainability” | “Food print” and “XXg CO_2_” | “Food print” and “good choice” |

**Supplementary Table 2.** Label type is based on the classification of Taufique et al. (2022)
